# Why Is Zeaxanthin the Most Concentrated Xanthophyll in the Central Fovea?

**DOI:** 10.3390/nu12051333

**Published:** 2020-05-07

**Authors:** Justyna Widomska, John Paul SanGiovanni, Witold K. Subczynski

**Affiliations:** 1 Department of Biophysics, Medical University of Lublin, Jaczewskiego 4, 20-090 Lublin, Poland; 2 Department of Nutritional Sciences, The University of Arizona, 1657 East Helen Street, Tucson, AZ 85721, USA; 3 Department of Biophysics, Medical College of Wisconsin, 8701 Watertown Plank Road, Milwaukee, WI 53226, USA; subczyn@mcw.edu

**Keywords:** lutein, zeaxanthin, macular xanthophyll, lipid bilayer, macula, AMD

## Abstract

Diet-based xanthophylls (zeaxanthin and lutein) are conditionally essential polar carotenoids preferentially accreted in high concentrations (1 mM) to the central retina, where they have the capacity to impart unique physiologically significant biophysical biochemical properties implicated in cell function, rescue, and survival. Macular xanthophylls interact with membrane-bound proteins and lipids to absorb/attenuate light energy, modulate oxidative stress and redox balance, and influence signal transduction cascades implicated in the pathophysiology of age-related macular degeneration. There is exclusive transport, sequestration, and appreciable bioamplification of macular xanthophylls from the circulating carotenoid pool to the retina and within the retina to regions required for high-resolution sensory processing. The distribution of diet-based macular xanthophylls and the lutein metabolite meso-zeaxanthin varies considerably by retinal eccentricity. Zeaxanthin concentrations are 2.5-fold higher than lutein in the cone-dense central fovea. This is an ~20-fold increase in the molar ratio relative to eccentric retinal regions with biochemically detectable macular xanthophylls. In this review, we discuss how the differences in the specific properties of lutein and zeaxanthin could help explain the preferential accumulation of zeaxanthin in the most vulnerable region of the macula.

## 1. Introduction

**Figure 1:**
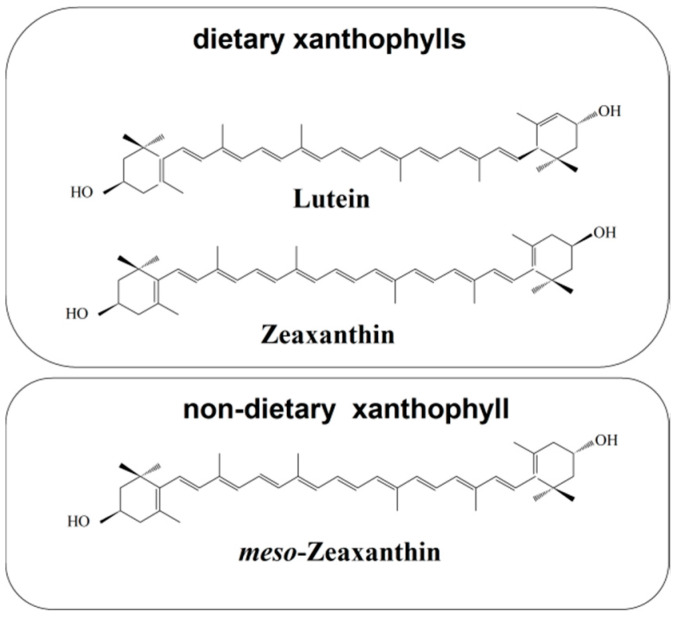
Chemical structures of macular xanthophylls present in the retina, including dietary xanthophylls (lutein and zeaxanthin) and non-dietary xanthophyll (meso-zeaxanthin). Retinal meso-zeaxanthin is a product of the conversion of lutein. This xanthophyll is rarely encountered in the human diet. However, meso-zeaxanthin may be absorbed after oral administration and transported to the retina [11].

Humans cannot synthesize carotenoids de novo and must acquire them through their diet. The most abundant carotenoids in human serum are three nonpolar carotenoids (α-carotene, β-carotene, and lycopene) and three polar carotenoids (β-cryptoxanthin, lutein, and zeaxanthin) [1,2]. The low serum zeaxanthin concentration contrasts with relatively high serum β-cryptoxanthin and serum lutein concentrations [1,2]. Polar serum carotenoids (xanthophylls) are the major class of carotenoids found in the brain and account for 70% of the total brain carotenoid content [3,4,5]. Only two carotenoids, lutein and zeaxanthin, are selectively accumulated in the retina and constitute 100% of the total retina carotenoid content [6,7]. Additionally, one of the stereoisomers of zeaxanthin, namely meso-zeaxanthin, is produced directly in the retina through the transformation of lutein [8]. The conversion of lutein to meso-zeaxanthin requires the migration of one double bond in the ε-ring of the lutein molecule and, most likely, this process takes place in the retinal pigment epithelium (RPE)/choroid [9,10]. All-trans zeaxanthin and meso-zeaxanthin are xanthophylls of the central part of the macula, whereas all-trans lutein dominates in the peripheral macula [7]. See Figure 1 for their structures.

Since primates cannot biosynthesize lutein or zeaxanthin [12,13], they have adapted mechanisms for efficient uptake [14,15], transport [16,17], retention [18,19,20,21,22], and repair [23]. The physiological significance of macular xanthophylls in retinal health and disease is supported by (i) their structural chemistry and inextricably linked biophysical properties [24], and (ii) the specific (a) accretion from a pool of approximately 40 dietary [25] and 15 circulating carotenoids [26,27], (b) laminar and topographic distribution in the retina [28,29,30,31,32], and (c) membrane disposition [33]. We have provided overviews on strong inferences linking macular xanthophylls to human retinal health through at least five converging lines of evidence [34,35,36]. In addition to the 1000–10,000-fold bioamplification of macular xanthophylls [24,37] and active transport [24] mechanisms involving specific binding proteins [14,16,17], there are works linking: (i) macular xanthophylls intake to primate retinal cellular and laminar macular xanthophylls status and structure through biochemical [7,38,39,40,41], ex vivo [23,30,31,37,42,43] and in vivo [13,37,44] analyses, (ii) macular xanthophyll supplementation to retinal structure and function in model rodent [45] and primate [12,46] systems and in human post-mortem [40] and clinical trials, and (iii) increased macular xanthophylls intake [47,48,49,50,51] and status [52,53,54,55] to advanced age-related macular degeneration (AMD). Substantial inter-individual variation exists in global [42] and local [56,57,58] topographic macular pigment density (MPOD) [21,59,60,61]. Sharifzadeh et al. describe five major patterns in macular pigment distribution within elderly people as measured by resonance Raman spectroscopy [62] and two wavelength autofluorescence [63] imaging techniques. In the Sharifzadeh cohorts, very low foveal MPOD existed in 10% of those studied, 20% of people showed a slightly enhanced foveal MPOD with macular xanthophylls extending to eccentric regions, 30% expressed a “sole, sharp, central distribution” of MPOD, 20% manifested a dense foveal MPOD with a ring of pigment surrounding this area, and 10% expressed a “uniform, laterally extended distribution” of MPOD.

For the purpose of this review, we highlight the fact that the distribution of specific macular xanthophylls varies with retinal eccentricity. Zeaxanthin and meso-zeaxanthin dominate in the fovea with concentrations declining more rapidly than those of lutein as distance from the fovea increases [7,39]. A 1° angular subtense in the retina represents ~0.29 mm of retinal extent. The zeaxanthin-to-lutein ratio at 0–5° from the fovea is approximately 1.5:1.0. At 5–19°, the value is approximately 1.0:1:5. At 19–38° the value is approximately 1.0:2.0 [7,39]. In 2005, Johnson et al. identified lutein as a dietary precursor of meso-zeaxanthin [12]. Although meso-zeaxanthin is virtually nonexistent in the human food supply and plasma, it is found at concentrations similar to zeaxanthin in the foveola (and shows a negligible signal outside of the fovea) [14]. The relatively lower concentration of lutein within the fovea has been used to suggest meso-zeaxanthin may be metabolized from oxidized lutein via a cone-photoreceptor-specific enzyme [7,39,64,65]. Thus, zeaxanthin is the dominant macular xanthophyll in the fovea center, an area with a high density of cone photoreceptors that is exposed to bright light. Noting that the human diet is zeaxanthin-poor, and that lutein is converted to meso-zeaxanthin in the retina, the following question arises: Why are isomers of zeaxanthin, not lutein, selectively accumulated in the central part of the fovea? The presence of oxygen in xanthophyll molecules explains their selective attendance in the retina and brain tissues because these two hydroxyl groups are responsible for the specific location and orientation of macular xanthophylls in the lipid bilayer. There is no difference in the orientation of macular xanthophylls in the lipid bilayer; both lutein and zeaxanthin adopt a similar transmembrane orientation [66]. A recent publication from Gruszecki’s group corrected their previous results, which indicated that zeaxanthin adopts mainly vertical orientation with respect to the plane of the membrane, whereas two pools, with vertical and parallel orientation, were proposed for lutein [67]. Also, our electron paramagnetic resonance spin-labeling studies have shown similar effects of lutein and zeaxanthin on the physical properties of the lipid bilayer, supporting the transmembrane orientation of these two macular pigments [68,69,70].

Not only is the orientation in the lipid membrane similar for lutein and zeaxanthin molecules (as discussed previously [66,68,69,70]), membrane distribution and location also are roughly the same. Our investigations, which were conducted using membrane models of photoreceptor outer segments and on lipid bilayers made of raft-forming mixtures, indicate that both macular xanthophylls are more concentrated in the unsaturated bulk domain (enriched in polyunsaturated lipids) and excluded from the raft domain (enriched in saturated lipids and cholesterol) [33,71,72]. Also, the macular xanthophyll–lipid interaction is not different for lutein and zeaxanthin [69,73]. Both increase the order of phospholipid membranes, decrease alkyl-chain motion in the fluid phase membranes, and increase the hydrophobicity of the membrane interior. Thus, there is still a need to explain why zeaxanthin is selectively accumulated in the foveola although its serum concentration is lower than that of lutein. The present paper aims to call this into question.

## 2. Zeaxanthin and Human Nutrition

### 2.1. Dietary Sources of Zeaxanthin

**Figure 2:**
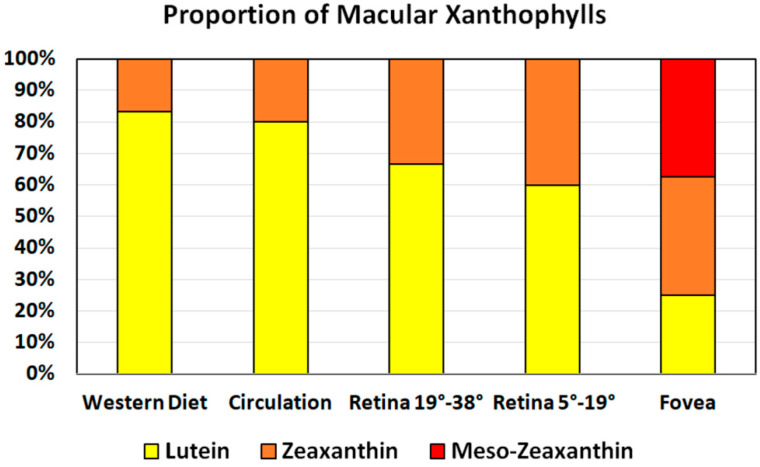
Proportion of major xanthophyll carotenoids in commonly consumed foods, circulation, and retinal areas, showing preferential accumulation of isomers of zeaxanthin in the fovea.

Dietary macular xanthophylls are found naturally in a wide variety of fruits and vegetables [74]. However, scientific papers usually report lutein and zeaxanthin content as one figure, not separately. Most food sources contain mainly lutein, which means that the human diet is poor in zeaxanthin. Dietary intake of zeaxanthin is much lower than that of lutein, with a dietary zeaxanthin-to-lutein molar ratio of 1:12 to 1:5 [75,76,77]. In human serum, the zeaxanthin-to-lutein molar ratio ranges from 1:7 to 1:4, which is consistent with the relatively high lutein content in fruits and vegetables as compared with the content of zeaxanthin [4,75,78,79]. Low zeaxanthin content in the daily human diet and in human serum contrast with the high concentration of this pigment in the fovea (Figure 2) where zeaxanthin-to-lutein molar ratio is 2.4:1 in the central part [8].

Corn and egg yolks contain approximately the same content of both macular carotenoids; the molar ratio of zeaxanthin-to-lutein is about 1 in both foods. Few foods contain more zeaxanthin than lutein. One food with a higher zeaxanthin-to-lutein molar ratio is the orange pepper, with a zeaxanthin-to-lutein molar ratio of approximately 10. Another such food is the fruit of the *Lycium barbarum*, commonly known as goji berries or wolfberries. Goji berries are the richest source of zeaxanthin (Table 1) and are widely used in traditional Chinese medicine for eye health. Daily supplementation with goji berries for 90 days increases serum zeaxanthin and in early AMD patients; goji berries can be used to prevent the progression of AMD [80]. Another rich source of zeaxanthin is the fruit of *Physalis alkekengi*, or the Chinese lantern [81,82], wherein zeaxanthin comprises more than one-half of the total carotenoid content. The berries of the sea buckthorn also have zeaxanthin ester as major compound, with zeaxanthin dipalmitate comprising up to 38% of the total carotenoid content and a zeaxanthin-to-lutein molar ratio of about 10 [83,84]. Despite the fact that eggs have a low zeaxanthin content compared with zeaxanthin-rich berries, the egg yolk provides an excellent dietary source of zeaxanthin because the bioavailability from the yolk matrix is much higher than from the leaves of green vegetables. The high bioavailability of a fat-soluble nutrient such as zeaxanthin from the egg is due to the rich lipid matrix of the yolk. Egg yolk is a good dietary source of both zeaxanthin and lutein, particularly as part of a typical western diet, which is poor in vegetables and fruits. It was reported that egg supplementation may increase plasma zeaxanthin by 142% [85]. Finally, it should be mentioned here that a high intake of lutein can also increase the macular content of meso-zeaxanthin because the lutein can convert to meso-zeaxanthin in the central retina.

### 2.2. Bioavailability of Zeaxanthin from the food Matrix

Zeaxanthin is a lipophilic molecule, which means that it is generally soluble in fat and insoluble in water. However, xanthophylls including zeaxanthin have lower lipophilicity compared with the carotenes present in human serum, and the facility (ease) of its release into an aqueous environment is probably higher than that of β-carotene and lycopene. Zeaxanthin must be released from the food matrix, which is an easier process with regard to the location of this pigment in the chromoplast of fruits rather than in the chloroplast of the leaves of green vegetables. In addition to its intracellular location, other factors strongly affect the bioavailability of zeaxanthin [86,87,88,89]; these include the (i) physical properties of carotenoids, (ii) food processing conditions, (iii) food sources and matrix in which zeaxanthin is incorporated, and (iv) co-presence of different compounds in the food, especially other carotenoids.

In nature, zeaxanthin exists in both free and esterified forms. Free zeaxanthin can be found in egg yolks [90], corn, and green vegetables, whereas flowers and fruits contain mostly zeaxanthin esters [91]. Most zeaxanthin esters in food exist in the form of zeaxanthin dipalmitate [92]. However, only the unesterified form has been detected in human serum and the retina, which means that esterified zeaxanthin must be hydrolyzed before it is incorporated into chylomicrons. It has been shown that the esterified form of zeaxanthin has higher bioavailability than the nonesterified form [93]. Breithaupt and colleagues reported [94] that zeaxanthin suspended in yogurt drinks and administered to volunteers was better absorbed in the esterified form than in the free form. Similarly, increased or comparable bioavailability of other xanthophylls as either the free or esterified form was reported [91,95,96,97].

These studies suggest that hydrolysis of the esterified form of zeaxanthin in the intestinal lumen is a highly efficient process.

Similar to other carotenoids in plants, zeaxanthin exists predominantly in the all-trans configuration, which is more stable thermodynamically as compared with the cis configuration. However, light [98] and thermal food processing [99] may increases the content of cis isomers. Both geometrical isomers of zeaxanthin (cis and trans) were detected not only in food sources but also in serum and retinal tissues. No research has been published about the bioavailability of the cis isomers of zeaxanthin. However, it has been indicated that cis isomers of lycopene have higher bioavailability than all-trans isomers [100]. Cis β-carotenes, on the other hand, exhibit lower absorption efficiency than all-trans β-carotene [101,102], which may be related to isomerization of cis β-carotenes to all-trans configuration in the erythrocyte.

Another physical factor affecting the bioavailability of zeaxanthin is its aggregation. In aqueous systems, zeaxanthin may form two aggregate arrangements: J-zeaxanthin (head-to-tail aggregates with characteristic red-shifted absorption) and H-zeaxanthin (card-pack aggregates with characteristic blue-shifted absorption). Hempel et al. reported [103] that the bioavailability of J-aggregates of zeaxanthin dipalmitate formulation was 23% higher than that of the H-aggregates of zeaxanthin but that the effect was marginally significant. Generally, the bioavailability of carotenoids is greater in heat-processed foods than in raw vegetables. The same relationship has been observed for zeaxanthin [104,105,106]. However, very high temperatures may lead to the degradation of carotenoid pigments and reduce zeaxanthin content by about 30%, as has been reported for corn canned at 121 °C [107]. The loss of zeaxanthin was also observed in fried and boiled eggs [108].

The next critical factor that should be considered as affecting the bioavailability of zeaxanthin is the food matrix in which the pigment molecules are incorporated. The release zeaxanthin from the food matrix is an easier process for simple food matrices, such as oil matrices, than it is for complex plant systems. Indeed, it has been shown that the xanthophylls were highly bioaccessible from fruits, ranging from 50 to 100%, compared with dark green vegetables, ranging from 19 to 38% [93].

The last factor affecting the bioavailability of zeaxanthin is the co-presence of different compounds in the food matrix. Several studies reported that the co-presence of the other carotenoids may affect the bioavailability of zeaxanthin. A daily oral dose (10 mg) of lutein provided a fourfold increase in lutein serum concentration as well as an increase in the amount of zeaxanthin [109]. In several studies, a competitive interaction was observed between lutein and β-carotene [110,111,112]. Similarly, a competitive interaction exists between β-carotene and zeaxanthin [110]. In a study of zeaxanthin bioavailability in chicks, the chicks’ diet was supplemented with high β-carotene; this led to decreased concentrations of both lutein and zeaxanthin in the plasma and most tissues, including the retina [110]. This competitive inhibition of certain carotenes and macular xanthophylls should be considered during supplementation with a carotenoid mixture.

The release of zeaxanthin from the food matrix starts in the stomach by the action of gastric acids and digestive enzymes. Next, molecules of zeaxanthin solubilized in lipid emulsion particles are transported from the stomach to the duodenum. Studies on the behavior of zeaxanthin in biological emulsion have shown that this dipolar molecule resides on the surface of oil droplets, together with proteins and phospholipids [113]. Conversely, a nonpolar carotenoid such as β-carotene is buried in the core of lipid droplets [113]. In the duodenum, the lipid emulsion particles with zeaxanthin are further stabilized by the addition of bile salts and phospholipids [114]. The bile acid reduces the size of the emulsified lipid droplets. The localization of zeaxanthin on the phospholipid surface of the emulsion influences the next step, which is the transfer of these molecules from the emulsified lipid drops to mixed micelles. It is widely accepted that xanthophylls are more easily micellarized then nonpolar carotenoids. Also involved in this transfer are both carotenoid polarity and bile lipid concentration and physiological variation in pH [115]. Bile salt micelles containing zeaxanthin must then be transported to enterocytes. Micelles are passed through the unstirred water layer and xanthophylls uptake is likely facilitated by scavenger and/or other lipid transporters of the apical membrane of enterocytes [116,117]. Once absorbed into the enterocytes, zeaxanthin is packed into lipoprotein particles (chylomicrons), which are released by exocytosis to the lymphatic system. Next, the zeaxanthin-chylomicron fraction enters the liver where it is stored or secreted back into the bloodstream to be transported to different tissues. Previous studies on the carotenoid–lipoprotein complexes have shown that nonpolar carotenoids are transported predominantly by LDLs (low density lipoproteins), whereas polar carotenoids were found equally in HDLs (high density lipoproteins) and LDLs. However, Thomas and Harrison [118] demonstrated that zeaxanthin is transported mostly by HDL lipoproteins. The literature suggests that the segregation of polar and nonpolar carotenoids already occurs on the level of carotenoid transport. Moreover, Connor et al. [119] demonstrated that HDL deficiency in the mutant chicken was associated with deficiency of lutein in the retina. In chicks fed a high-lutein diet, the content of lutein greatly increased in the plasma, liver, and heart, but increased only a little in the retina [119].

## 3. Zeaxanthin in the Human Retina

### 3.1. Mechanisms of Selective Transport

The uptake and transport of macular pigment to the retina is considered selective because of the approximately 600 carotenoids identified in nature, 50 in the human diet, and 20 in human serum; only two forms of dietary xanthophylls, lutein and zeaxanthin, are present in the retina. Meso-zeaxanthin is the third major macular xanthophyll (reviewed in [86]). This selective delivery mechanism probably is the active transport process. As mentioned previously, both lutein and zeaxanthin are associated mostly with HDL in human serum. Recent in vitro studies have shown that zeaxanthin is more efficiently delivered to the human RPE cell model (differentiated ARPE-19) via HDLs whereas lutein is delivered via LDLs [118]. The circulating HDLs and LDLs must pass through the choroid and Bruch’s membrane before reaching the RPE, while in the neural retina, they must cross the capillary endothelial cells to enter Müller cells [120]. The major receptors for LDLs are the LDL receptors (LDLRs), whereas HDL is recognized by scavenger receptor class B type 1 (SR-BI) [121]. SR-BI is a cell surface glycoprotein with a high affinity to HDL and is involved in zeaxanthin uptake in RPE. [120]. LDLR receptors recognize apolipoprotein B and are responsible for the uptake of LDLs and cholesterol, and probably also lutein–LDL complexes [118], by the retina [122]. We have provided detailed commentary on the links between lipoproteins, scavenger receptor molecular genetics and retinal xanthophyll status in the context of AMD pathophysiology [34,36]. Shyam et al. demonstrated that while all three scavenger class B receptors (SRB: SR-B1, SR-B2, and CD36) present in the macula are capable of binding and transporting all three macular xanthophylls, the uniform expression profiles of these proteins in human macula and eccentric retinal regions suggests that selective xanthophyll-binding proteins (including StARD3 and GSTP1, K_D_ ~ 0.6 μM) are most likely to drive the process of selective accumulation of xanthophylls into the macula [123]. These authors used surface plasmon resonance analyses to demonstrate that all three scavenger class B receptors (SRB: SR-B1, SR-B2, and CD36) present in the macula bind macular xanthophylls with affinities characteristic of transport proteins (K_D_ ~ 1–2 μM). Macular xanthophylls were delivered in LDL and HDL complexes at physiologic concentrations to characterize distinct aspects of uptake for lutein, zeaxanthin and meso-zeaxanthin using a SRB receptor class over-expressing model cell system (HEK-293T) that does not endogenously express these proteins. LDL-bound lutein uptake was enhanced and mediated by SR-B1 and CD36 receptors. Zeaxanthin and meso-zeaxanthin delivered by HDL was taken up in appreciable amounts by cells overexpressing any of the three SRB proteins. SR-B2 over-expressing cells transported zeaxanthin at concentrations 10-fold to those for lutein. However, non-transfected (SRB-free) control cells took up higher amounts of zeaxanthin and meso-zeaxanthin than lutein, raising the possibility that selective accretion of zeaxanthins by these cells that may be SRB independent.

It is not clear whether macular xanthophylls are incorporated directly into the lipid-bilayer portion of retina membranes or are bound by membrane-associated xanthophyll-binding proteins. Three of the xanthophyll-binding proteins (xanthophyll transporters) have already been identified and characterized by Bernstein’s group. These are lutein-specific steroidogenic acute regulatory domain protein (StARD3) [124,125], zeaxanthin-specific glutathione S-transferases pi 1 (GSTP1) [17], and members of the tubulin family [126], which demonstrate weaker binding properties than StARD3 and GSTP1. The question is whether these proteins are only selective transporters responsible for the specific and different distribution of lutein and zeaxanthin in the retina or whether they can store macular xanthophylls in membranes.

**Figure 3:**
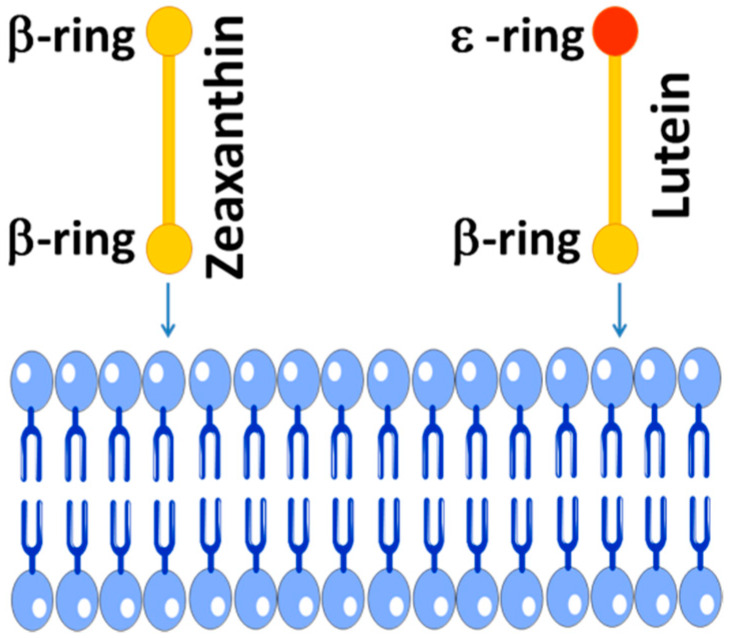
Schematic drawing showing the preferential lutein β-ring-intercalation into the lipid membrane based on molecular dynamics study of Makuch et al. (for more details, see Ref. [127]). Two macular xanthophylls are shown: symmetrical zeaxanthin with two β-rings and asymmetrical lutein with one β-ring and one ε-ring.

The zeaxanthin molecule is completely symmetrical compared with the asymmetrical lutein (Figure 1). Structurally, the main difference between lutein and zeaxanthin is the position of the double bond in the terminal ionone ring. The two rings of lutein, namely β-ring and ε-ring, are not equivalent, whereas the zeaxanthin molecule has two identical β-rings. Recently, Makuch et al. [127] showed that the intercalation of lutein into the lipid bilayer is also asymmetrical. Lutein with one β-ring and one ε-ring intercalates into the bilayer preferentially with its β-ring, as shown schematically in Figure 3. The asymmetric intercalation of lutein into the phospholipid bilayer coincidence with its asymmetrical docking into the active side of the enzyme that catalyzes the conversion of lutein into meso-zeaxanthin [9,10] and into the asymmetric cavity of the lutein binding protein [125]. In this asymmetrical docking, the β-ring stays outside the cavity. This asymmetrical intercalation of lutein with the lipid bilayer and xanthophyll-binding proteins may be the key to understanding differences in spatial distribution and transferring two xanthophylls to the retina.

### 3.2. Spatial Distribution

**Figure 4:**
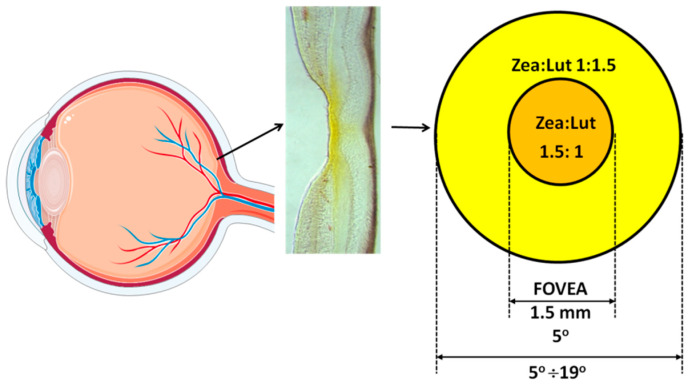
Vertical section of a monkey fovea showing the distribution of macular xanthophylls (yellow color). (Adapted with permission from Snodderly, D.M., 1995. Am J. Clin. Nutr. 62, 1448S-1461S [23].) The zeaxanthin-to lutein ratio at 0–5° is 1.5:1.0; at 5–19°, 1.0:1.5; and at 19–38°, 1.0:2.0 (a 1° angular subtense in the retina represents 0.29 mm of retinal extent) [7,39].

The center of the fovea [8,128] contains the highest zeaxanthin, and the zeaxanthin concentration declines to negligible levels outside 7° of the foveal eccentricity [37]. The zeaxanthin-to-lutein molar ratio changes as the eccentricity moves away from the fovea (Figure 4). At distances exceeding 5.5 mm of perifovea, the zeaxanthin-to-lutein molar ratio is between 0.5 and 0.3, whereas the ratio is reversed to 2.4 in the center of fovea [7]. The resented values include zeaxanthin and the isomer of zeaxanthin, meso-zeaxanthin.

Why meso-zeaxanthin is produced in the retina from lutein and so efficiently replaces lutein in the central part of the fovea is still an unexplained phenomenon. To a certain degree, the high presence of meso-zeaxanthin in the foveal region of primates can be explained by the primates’ common diet, which is low in zeaxanthin and rich in lutein (precursor of meso-zeaxanthin). This points out the need for the presence of this particular macular xanthophyll in the central macula. The concentration peak of meso-zeaxanthin in the central fovea correlates with the peak of the cones; one can speculate that meso-zeaxanthin is associated with cones. When plotted as a function of eccentricity, the zeaxanthin (including meso-zeaxanthin)-to-lutein ratio follows the same pattern as the cone-to-rod ratio; as such, it has been proposed that these two xanthophylls are associated with different types of photoreceptors, i.e., rods versus cones [7]. Another observation indicates that this hypothesis is insufficient and both lutein and zeaxanthin were isolated from retinal pigment epithelium and rod outer segments [31]. Thus, the association of the xanthophylls with the certain photoreceptor types is not straightforward.

The spatial distribution profile of macular pigment is measured by several physical techniques. Methods for macular pigment imaging include autofluorescence imaging, fundus reflection methods, resonance Raman spectroscopy, and fluorescence lifetime imaging ophthalmoscopy. Using the fundus reflectometry technique, MPOD can be determined by analysis of the reflectance of light from the foveal and perifoveal regions and the spectral contribution of the absorbance of xanthophyll molecules. The autofluorescence method measures the intensity of the autofluorescence of lipofuscin in the presence of excitation with two wavelengths, one fully and the other minimally absorbed by xanthophylls. The Raman spectroscopy method uses excitation of the carotenoids in their absorption band, which occur in the blue/green wavelength region, and detects signals from the stretch vibration of the carbon double bonds in part of the polyene chain of the carotenoid molecule. The above-motioned fluorescence lifetime imaging ophthalmoscopy method measures the lifetimes of retinal fluorophores, including the short lifetime of macular xanthophylls [129,130]. The in vivo assessment of the MPOD can also be obtained by psychophysical methods, such as heterochromatic flicker photometry and minimal motion photometry. Both subjective psychophysical tests and objective optical imaging methods show that the concentration of macular xanthophylls peaks toward the center of the fovea. HPLC (high-performance liquid chromatography) ex vivo studies of primate and human retinas confirm the spatial density distribution of macular pigment [131]. More recent evidence suggests that the anatomical characteristics and spatial distribution of Müller cells at the fovea and parafovea are associated with the distribution of macular pigment. The human retina contains three types of glial cells: microglia, astrocytes, and Müller cells. The fovea does not contain astrocytes and microglia; its structure contains only Müller cells. The fovea contains two different pools of Müller cells: Müller cells cones and z-shaped Müller cells of the foveal walls. Macular telangiectasia type 2 (MacTel 2) is an idiopathic bilateral retinal condition associated with vision loss and the redistribution of macular xanthophylls. MacTel 2 commonly presents in middle age and is characterized by degenration of the neural retina along with vascular changes altering structures in the outer nuclear layer|ellipsoid zone. End stages yield pseudolamellar macular holes. Hyperplasia and movement of RPE cells to inner retinal layers may occur in late stages. Hypertension and diabetes mellitus are associated with the condition. However, one of the earliest manifestations of MacTel type 2 is different than normal redistribution of macular xanthophylls, with the loss of macular xanthophylls in the center of the fovea. Gass et al. [132] proposed that Müller cell abnormalities play a crucial role in the retinal degeneration of MacTel type 2 and that Müller cells loss in MacTel type 2 may explain the observed lack of macular pigment in the foveola visible in the MPOD images. The correlation between areas of macular xanthophyll absence and areas of Müller cell depletion was observed also by Powner et al. [133,134].

### 3.3. Retinal Layers Distribution

In contrast with the spatial distribution of xanthophylls within the retina, information about the exact subcellular location of yellow pigments in different retinal layers is limited. An initial study of the distribution of xanthophylls in the human retina reported a similar relationship between the zeaxanthin-to-lutein molar ratio and cone-to-rod ratio with eccentricity [39], suggesting the association of specific xanthophyll with specific photoreceptors. However, Snodderly et al. [131] did not agree with the hypothesis that zeaxanthin is preferentially associated with cones and lutein with rods. First, Snodderly et al. showed that, in contrast with humans, the distribution of xanthophylls had a reveres pattern in squirrels and macaque monkeys, with more lutein than zeaxanthin throughout the central fovea. Second, Snodderly et al. indicated that lutein is present in the foveola (central part of the fovea) where only cones exist. Finally, it was shown that both macular xanthophylls were detected in rod outer segment membranes of the human retina [31,43]. Additionally, some carotenoids were isolated from the RPE [32]. However, the predominant localization of xanthophylls is the outer plexiform layer (Henle’s fiber layer) in the fovea and the inner plexiform layer in the parafovea [39,131]. A different approach of checking the retinal layer distribution of macular xanthophylls was demonstrated by Bernstein’s group, which reported the distribution of carotenoids in the retina layer through analysis of the distribution of retinal xanthophyll-binding proteins [14,17,32]. Two xanthophyll-binding proteins were found in the human retina: the zeaxanthin-binding protein GSTP1 and the lutein-binding protein StARD [14,17,32]. Immunocytochemistry with an antibody to GSTP1 showed the strongest labeling of the outer plexiform layer (Henle’s fiber layer) [17]. Immunolocalization with antibodies directed against StARD showed selective labeling of monkey photoreceptor inner segments [16]. Gass [132] first suggested that the Müller cell cones can form a reservoir of zeaxanthin. Macular Müller cells as the principle cellular reservoir of macular xanthophylls have received less attention over the last two decades because knowledge regarding foveal glial cells was not complete. In the fovea, Müller cells run parallel to Henle’s fibers [135] and diagonal to incoming light; this may give a misconception as to where exactly macular xanthophylls are located in the fovea (i.e., in Müller cells or Henle’s fibers). Initially, the predominant xanthophyll pigment was thought to be located in the Henle’s fiber layer [7,131,136], but Müller cells may also be the main site of xanthophyll location [132].

## 4. Antioxidant Properties of Zeaxanthin

### 4.1. Physical Antioxidant Actions

Zeaxanthin may quench singlet oxygen by a physical mechanism in which zeaxanthin can participate in multiple quenching cycles without degradation. During that physical process, the excess energy from singlet oxygen is transformed to carotenoid and into its excited triplet state. Next, the carotenoid-excited triplet state is deactivated through thermal decay. Retaining an intact structure in that physical process ensures that the zeaxanthin molecule can be reused, thus increasing the efficiency of the quenching process. The efficiency of physical quenching of singlet oxygen by carotenoids was reported to be greater than that of vitamin E [137] and is directly related to the conjugation chain length of the pigment molecule. In organic solvents, zeaxanthin (with 11 conjugated double bounds) has a higher singlet oxygen quenching rate constant than lutein (with 10 conjugated double bonds) [138]. Similarly, in model membranous systems, zeaxanthin exhibits a better ability to quench singlet oxygen than lutein [139]. It has been shown that oxidation initiated by the all-trans retinal derived retinal photosensitizer A2-PE (composed of two molecules of all-trans retinal and phosphatidylethanolamine) was suppressed more effectively in the presence of zeaxanthin (which is a better singlet oxygen quencher) than in the presence of lutein [140]. A2-PE is one of many retinal photosensitizers that creates harmful oxygen species when exposed to blue light.

In plants, the excited triplet state of chlorophyll (photosensitizer) is efficiently quenched by the carotenoid molecule, leading to heat dissipation and preventing the formation of singlet oxygen. It should be mentioned that the quenching of the excited triplet state of photosensitizers by xanthophylls requires a close distance approach of these two molecules. Triplet chlorophylls in the light-harvesting complexes are promptly quenched by lutein [141] and zeaxanthin [142] and bind to specific sites within light-harvesting antenna complexes. Thus, carotenoids can exert their physical antioxidant action not only by direct singlet oxygen quenching but also by transferring energy from the triplet states of sensitizers. It is accepted that some endogenous photosensitizers producing reactive oxygen species are present in the retina. In the physiological condition, all-trans retinal is converted to 11-cis retinal, causing rhodopsin activation. After that, all-trans retinal is released from photoactivated rhodopsin and recycled. However, many retinal degeneration processes may be induced by disrupting all-trans retinal clearance [143,144,145]. If the clearance process is not effective, all-trans retinal will accumulate in the rod outer segment, and light and the presence of oxygen will generate toxic oxygen species. Despite the fact that xanthophylls are capable of quenching excited triplet states of photosensitizers, physical antioxidant actions have not been experimentally confirmed for retinal xanthophylls and retinal endogenous photosensitizers.

Because of the presence of potent photosensitizers in the retina, much oxidation damage can be caused by short-wavelength light. Photosensitizers that can be activated by blue light produce singlet oxygen, which may be a risk factor for harmful oxidation, leading to AMD [146]. Both lutein and zeaxanthin absorb blue light, and this has led to the hypothesis that macular xanthophylls may protect the retina by filtering blue light [136,138] and prevent the formation of singlet oxygen. The prereceptoral location of macular pigment is ideal for this function. It has been reported that macular xanthophyll supplementation protected the fovea from blue-light-induced damage [46]. The anatomical distribution of zeaxanthin decreases with increasing eccentricity from the fovea. Thus, zeaxanthin acts as screening pigment for the underlying densely packed receptors. The optical absorption spectra of lutein and zeaxanthin are very similar. In the organic solvent, the absorption maximum of zeaxanthin and meso-zeaxanthin is 450 nm and the maximum of lutein is shifted only 5 nm to the shorter wavelength.

### 4.2. Chemical Antioxidant Actions

The chemical quenching of singlet oxygen by carotenoids involves a chemical reaction between oxygen and xanthophyll molecules and, in contrast with physical quenching, xanthophylls are consumed and destroyed in this chemical process. The chemical deactivation of singlet oxygen by carotenoids has been reported as a minor reaction as compared with physical quenching [147,148,149,150,151]. However, several singlet oxygen xanthophyll oxidation products were detected in retina tissue, indicating that this reaction takes place not only in vitro [32,152]. Potentially, each double bond in the carbon chain of carotenoids can be oxidized by singlet oxygen, leading to the formation of different aldehydes. In the case of xanthophylls, a low level of aldehydic oxidation product formation was observed [153]. Endoperoxides were identified as the primary oxidation products of the chemical quenching of singlet oxygen by zeaxanthin [151,153]. Moreover, Ramel et al. [153] showed that the rate of formation of zeaxanthin endoperoxide in the organic solvent was slower than the formation of β-carotene and lutein endoperoxides. Similarly, during the chemical quenching of singlet oxygen by zeaxanthin, aldehydic oxidation products were formed later than in the case of singlet oxygen quenching by lutein and β-carotene [153]. From these studies, one can conclude that zeaxanthin is less susceptible to oxidation by singlet oxygen than lutein and β-carotene. It should be mentioned that chemical quenching causes bleaching of carotenoids and leads to the loss of physical quenching abilities.

**Figure 5:**
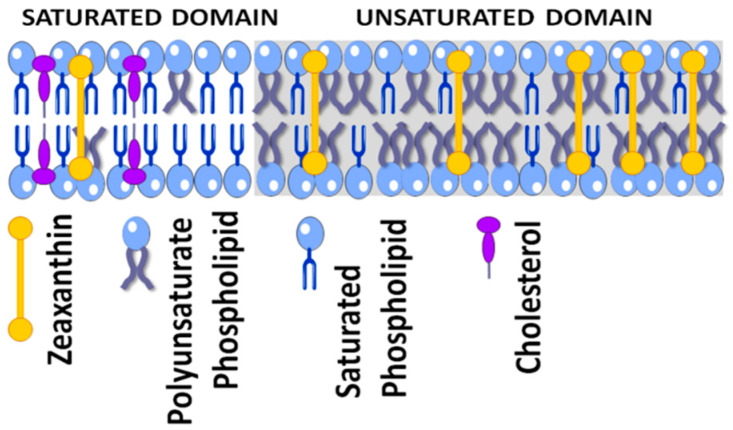
Schematic drawing showing the colocalization of zeaxanthin together with polyunsaturated phospholipid in the membrane domain. For more details, see Refs. [33,71,72].

Another sacrificing mechanism that occurs during protection of the retina is radical scavenging by lipid-soluble molecules of zeaxanthin. However, this action also causes zeaxanthin to lose its intact structure. It has been shown that zeaxanthin scavenged the hydroxyl radical more effectively than lutein [154], and the different distributions of lutein and zeaxanthin suggest that scavenging hydroxyl radicals is more important in the central fovea. Both retinal rods and cones of the outer segment disc membranes are very rich in long, polyunsaturated fatty acids [155,156], particularly docosahexaenoic acid, which is susceptible to oxidation. The selective localization of macular xanthophylls in domains rich in polyunsaturated phospholipids [33,71,72], and therefore susceptible to free-radical- and singlet-oxygen-induced damage, is ideal for this chemical antioxidant action. The phospholipids containing very-long-chain polyunsaturated fatty acids present in the disk membranes of rod outer segments likely play a unique and important role in the retina because they are necessary for cell survival and their loss leads to cell death [157,158]. Additionally, epidemiological studies of long-chain polyunsaturated phospholipid intake suggest a protective role against the incidence of advanced AMD [159,160]. Our investigations, made on a lipid model of photoreceptor outer segment membranes, indicate that xanthophylls were about 14 times more concentrated in the unsaturated bulk domain (enriched in polyunsaturated lipids) and excluded from the domain enriched in saturated lipids and cholesterol [71]. A similar distribution also was found in membranes made of a raft-forming mixture [72] where macular xanthophylls lutein and zeaxanthin were about eight times more concentrated in the bulk, unsaturated domain than in the saturated raft domain (Figure 5).

## 5. Zeaxanthin Supplementation

### 5.1. Zeaxanthin Supplements and AREDS Formulation

AMD is a chronic, complex degenerative disease that affects the macular region and may result in a loss of central vision. Oxidative stress is implicated in the pathogenesis and pathophysiology of AMD. The Age-Related Eye Disease Study 2 (AREDS2) [161] was a phase III randomized controlled trial designed to evaluate efficacy and safety of formulation containing dietary retinal xanthophylls and long-chain polyunsaturated fatty acids for the prevention progression to advanced AMD in people at moderate to high risk for the condition. This work built on the findings from the Age-Related Eye Disease Study (AREDS) [162,163]. In AREDS, participants were randomly selected to receive daily oral tablets in a factorial design for one of four treatments containing: (i) vitamins with antioxidant properties alone (500 mg vitamin C, 400 international units vitamin E, 15 mg β-carotene), (ii) zinc alone (80 mg zinc as zinc oxide along with 2 mg copper as copper oxide to prevent pernicious anemia), (iii) a combination of vitamins with antioxidant properties and zinc, or (iv) placebo. In this study, participants randomized to vitamins with antioxidant properties + zinc were 25% less likely than participants on placebo to progress to advanced AMD [156,157]. Analysis of AREDS dietary data showed that people reporting higher levels of dietary lutein and zeaxanthin [35] and omega-3 fatty acids [35,159,164,165] were also less likely to develop sight-threatening AMD [153,160]. A primary goal of the AREDS2 study was to determine if the addition of macular xanthophylls and/or omega-3 long-chain polyunsaturated fatty acids (LCPUFAs) to the original AREDS formulation would further reduce the risk of progression to advanced AMD and cataract in people at moderate to high risk for these events. The commercial AREDS2 formulation contains zinc plus vitamins from the AREDS formulation including macular xanthophylls (500 mg vitamin C, 400 international units vitamin E, 80 mg zinc as zinc oxide, 2 mg copper as cupric oxide, 10 mg lutein, 2 mg zeaxanthin) [161,166,167]. The choice to replace β-carotene in the original AREDS formulation with zeaxanthin and lutein was based on: (i) changes in availability of supplement-grade zeaxanthin and lutein (major endogenous retinal carotenoids) in quantities that would support the needs of the study; and, (ii) the demonstration in phase III trials that smokers taking supplements with β-carotene were more likely than their peers to develop lung cancer [168]. In addition to electively taking the AREDS supplements, participants in AREDS2 study were randomly assigned to receive one of four treatments: (i) 10 mg lutein + 2 mg zeaxanthin/d; (ii) omega-3 LCPUFAs, specifically docosahexaenoic acid (DHA, 350 mg/day) and eicosapentaenoic acid (650 mg/day); (iii) both lutein and zeaxanthin with LCPUFA; or (iv) control (no xanthophylls and no omega-3 LCPUFAs). The AREDS2 product was commercialized and is now recommended for individuals with intermediate to advanced AMD, based on the AREDS2 results (see Table 2). In AREDS2, comparisons of participants randomized to the xanthophyll-containing formulation to their peers not randomized to the xanthophyll formulation showed reduced likelihood of progression to advanced AMD. Analysis of participants reporting low dietary intake of lutein and zeaxanthin showed stronger associations; this draws attention to the fact that xanthophyll supplementation is very important in a low xanthophyll diet. AREDS formulation did not further reduce risk of progression to advanced AMD. A follow-up work on the AREDS2 applying post-hoc exploratory comparisons of lutein/zeaxathin interventions to those with β-carotene indicated that people in the macular xanthophyll arm were approximately 20% less likely than people in the β-carotene arm to develop late AMD or neovascular AMD. Results did not attain significance for this xanthophyll v. β-carotene comparison on the central geographic atrophy endpoint. Results persisted when analytic cohorts were restricted to people with moderate AMD at enrollment [169].

In the AREDS2 formulation, the dominant carotenoid is lutein. Similarly, most of the vision supplements commercially available primarily contain high amounts of lutein and low amounts of or no zeaxanthin. This decision was made based on the distribution of zeaxanthin and lutein in foods. For supplement production, zeaxanthin can be obtained from microbial sources [170,171,172] or by synthesis [173,174,175]. Most commercially available lutein/zeaxanthin products are extracted from marigold flowers [176,177]. Because most AREDS2-like supplements on the market are made by extracting plant oleoresins from the marigold, most contain high lutein content and low zeaxanthin content. Commercially available marigold supplements contain lutein and zeaxanthin in the molar ratio of 5:1 to 20:1 [178,179]. Keeping in mind that the ratio of zeaxanthin-to-lutein in the fovea is approximately 1.5:1 (at 0–5°), one should consider whether increasing the content of zeaxanthin in supplements for eye health will more effectively reduce the risk of AMD than lutein-rich supplements.

### 5.2. Effects of Zeaxanthin Supplementation on MPOD

The content of macular xanthophylls in the retina cannot be measured directly. However, the amount of retina carotenoids is related to MPOD. MPOD is the highest in the central fovea and decreases rapidly with increasing eccentricity [7]. It has been shown that supplementation with xanthophylls increases MPOD [20,37,180,181,182], and it is widely accepted that low MPOD might be a risk factor for AMD progression [48,51,183,184,185]. The effects of macular xanthophyll supplementation on MPOD were primarily studied using only lutein supplementation [186,187] or high-dose lutein and low-dose zeaxanthin supplements (with a zeaxanthin-to-lutein molar ratio ranging from 1:33 to 1:5) [20,177,178,179,180]. Conversely, the effect of high-dose zeaxanthin supplementation on MPOD is less studied. High-dose supplementation was studied by Bone et al. In this study, a high zeaxanthin dose (30 mg/day) was less absorbed than an equal lutein dose. In another study [188], a lutein dose (20 mg/day) increased MPOD more effectively than a lower dose (10 mg/day) in the short term (48 weeks) but had the same effect in the long term (two years). Both low and high lutein doses were more effective in increasing MPOD than combined supplementation with lutein (10 mg/day) and zeaxanthin (10 mg/day).

In MacTel type 2, macular xanthophylls have characteristic distribution with the depletion of xanthophylls from the central part of the fovea and their accumulation in the fovea surrounding ring. Unfortunately, in patients diagnosed with MacTel type 2, the lutein/zeaxanthin supplementation did not increase the xanthophyll content in the central fovea, the area where the macular xanthophylls were previously absent [22,189].

## 6. Conclusions

The biophysical and biochemical properties of macular xanthophylls support inferences on their physiologic significance. Membranes of photoreceptors containing polyunsaturated fatty acids susceptible to photo-oxidation exist in the retina at high oxygen tension and under chronic light exposure. Several vitamins with antioxidant properties and other antioxidants are implicated as protective agents in the retina, and these include vitamin E [190,191,192], vitamin C [49,190], selenium [193,194], glutathione [195], some enzymes [196,197,198,199], and two diet-based xanthophylls (lutein and zeaxanthin). However, we must consider the adaptive significance of the specific cellular and laminar spatial distribution of these two xanthophylls. It should be noted that both macular xanthophylls and vitamin E exhibit nonuniform spatial distribution within the macula [23]. Some explanation of the unique position of zeaxanthin in the protection of the retina comes from studies conducted on the thylakoid membranes of higher plants. Similar to photoreceptor membranes, these membranes are rich in polyunsaturated fatty acids, exist at high oxygen tension, and are exposed to intensive light. Zeaxanthin-deficient plants exhibit strongly reduced tolerance to oxidative damage, while zeaxanthin-overexpressing plants exhibit a strongly increased tolerance to oxidative stress [200,201,202]. Thus, during evolution, zeaxanthin conferred an advantage in effectively protecting thylakoid membranes.

Several factors explain how zeaxanthin (and meso-zeaxanthin) can be concentrated to a greater extent than lutein within the fovea. First, the presence of two functional ionone hydroxyl groups at the ends of this molecule determines its transmembrane orientation, which enhances the stability of zeaxanthin in the lipid bilayer membranes [68,70,203]. Second, the high antioxidant capacity of zeaxanthin is enhanced by the fact that it is less susceptible than lutein to consumption and destruction during chemical singlet oxygen quenching [153]. Third, zeaxanthin possess a higher capacity of singlet oxygen physical quenching than lutein [138,140]. All of these ensure that zeaxanthin efficiently protects against the oxidative stress that may lead to retinal damage.

As mentioned in Section 2.1, the human diet contains 5 to 12 times more lutein than zeaxanthin; lutein is found in higher quantities in human serum, which is likely due to the xanthophyll composition in foods. These proportions are reversed in the central part of the fovea where the concentration of zeaxanthin is about two times greater than the concentration of lutein—each xanthophyll has a retina-resident active transporter—and we have provided published reviews on genetic variation and macular xanthophyll transport and metabolism. At the moment, there is an unmet need to address the question formulated in the title of this review: “Why is zeaxanthin the most concentrated xanthophyll in the central fovea?” Although we have provided insights on differences in the selective transport of lutein and zeaxanthin from food to the retina and identified differences on the level of xanthophyll-binding proteins (which can ensure the specific and different distribution of lutein and zeaxanthin in the retina), the exact mechanisms driving the preferential accretion of zeaxanthin in the foveola remain unidentified.

Based upon our review of the evidence, it is our opinion that, although some physical and chemical properties of lutein and zeaxanthin are similar, the distinct properties of zeaxanthin offer an advantage for modulating processes implicit in the pathogenesis of AMD. These differences are not vast, but they are significant. Major differences include greater chemical stability [153], more effective physical quenching of singlet oxygen [138,140], and easier incorporation to lipid bilayer membranes [127]. We believe these differences emerged during evolution, and that zeaxanthin offered an adaptive advantage to protect the central, most vulnerable part of the retina. We conclude with the observation that the natural history of AMD will often lead to a sparing of the zeaxanthin-rich foveola as the last functional retinal region remaining prior to the loss of central vision.

## Figures and Tables

**Table 1:** Dietary sources of zeaxanthin.

Food	Zeaxanthin Content (μg/100 g)
Goji berry	280,000
Red Chinese lantern fruit	84,700
Orange pepper	5580
Sea buckthorn	1930
Egg yolk (raw)	762
Corn	105
Orange juice	26
Peach	39
Spinach	75
Kale	62
Papaya	6

**Table 2:** AERDS formulations.

AREDS1	
Vitamin C	500 mg
Vitamin E	400 IU
β-carotene	15 mg
Zinc	80 mg
Copper	2 mg
**AREDS2**	
Vitamin C	500 mg
Vitamin E	400 IU
Zinc	25 mg
Copper	2 mg
Zeaxanthin	2 mg
Lutein	10 mg
